# Heads of Clinical Observations on Mistakes and Oversights in the Treatment of Strangulated Hernia

**Published:** 1834-01-01

**Authors:** Herbert Mayo

**Affiliations:** Surgeon to the Middlesex Hospital.


*Heads of Clinical Observations on Mistakes and Oversights in the Treatment of Strangulated Hernia.* By Herbert Mayo, f.r.s., Surgeon to the Middlesex Hospital.

In strangulated hernia, the first object of the surgeon is to relieve his patient without the pain and risk attendant on an operation. He looks therefore anxiously for the abatement of one or other of the symptoms, during the preliminary treatment, by bleeding, the warm bath, injections, and the taxis. But some of the symptoms are liable to be *delusively* ameliorated, while the patient in reality is either stationary or growing worse. For instance, *diminution in tenseness and volume of the tumour, or, under certain circumstances, its absolute disappearance*, may possibly take place, while the stricture and the obstruction of the bowels remain unremoved.

I have repeatedly seen inguinal hernial tumours become less in volume, and less tense, without the urgency of the more important symptoms being suspended, through the water of the hernial sac being forced by the pressure used into the abdominal cavity. When the operation has been subsequently performed, the serum, which has been forced into the abdomen, has poured out through the neck of the sac, upon the division of the stricture.

I recollect seeing the body of a female examined who had died of crural hernia. She was not my patient; but the surgeon, under whose care she had fallen several hours after the invasion of symptoms of strangulation, had resorted to the taxis; he had compressed the tumour till it had disappeared, and in his opinion had been reduced. A small segment of the circumference of the bowel, however, had remained nipped in the sac, and caused death. Whether the diminution and supposed return of the tumour in this case was the emptying of its serum into the belly, or the actual reduction of a part of the protruding bowel, I had no means of ascertaining.

The following case I did not witness, but I was assured it happened as I shall describe. A patient laboured under strangulated inguinal hernia: the symptoms not being very urgent, and the tumour not very tender, considerable pressure was used, and at length the greater part of the tumour disappeared. The patient, however, was not much better; became worse, and died. Upon examination, it was found that the bowel had indeed been pushed back, *but that the sac had also been pushed back with it;* the neck of the sac, formed of thickened peritoneum, had been sufficient to keep up the strangulation.

A patient (a recent case in the Middlesex Hospital,) had all the symptoms of strangulated hernia: there was a small tumour, feeling like an omental hernia, at the crural arch. The patient had a swollen and tender belly, and stercoraceous vomiting. Repeated attempts had been made to reduce the rupture, which the patient said was considerably larger before these attempts had been used. The bowels had acted twice with enemata. I did not attempt to return the tumour, but operated immediately, when I found an empty sac: I divided the neck of the sac. The patient died in thirty hours. On opening the abdomen, the upper part of the small intestine was found distended, swollen, and inflamed. A segment of a portion of the ileum, which had been down, was deeply discoloured, and retained the impression of the close grip of the neck of the sac. It had been forced back into the belly before the performance of the operation, by the taxis, too much injured for recovery, through the length of time it had been strangulated. The tumour upon which I operated was the sac, with a thick band of adipose substance partially surrounding it.

Another symptom in strangulated hernia, which occasionally but very rarely occurs, and is the more likely to prove delusive, is *the action of the bowels either not being suspended, or returning:* this circumstance is not incompatible with total obstruction.

A patient was admitted into the Middlesex Hospital, with stercoraceous vomiting, and with frequent purging. His belly was swollen, and tense, and tender; and there was a large swelling of the right side of the scrotum and groin. The lower part of the swelling was distinctly a hydrocele; but the upper part, though to all appearance it was continuous with the tumour, I thought, must be an inguinal hernia: it had little tension and tenderness, and hardly any impulse was communicated to it on coughing. The *purging continued,* and the stercoraceous vomiting, and the patient grew worse. On the second day I operated: it was too late, there was a portion of intestine strangulated. The patient died of the peritonitis which it had occasioned.

The preceding facts suggest obvious but important rules, as to the period in strangulated hernia during which the taxis may be employed, and the degree of pressure which may be safely exerted, and strongly exemplify the danger of delay in operating, when only a seeming good is being attained by the other measures put in practice. It is very certain that more cases are lost by the delay of the operation than are endangered by its unnecessary performance.

The cases in which I have either seen the operation unnecessarily performed, or have had reason to be satisfied that I opposed the wish for an immediate operation, have been cases of omental hernia.

There are two reasons for operating in omental hernia: first, although a surgeon is sure to know by the touch when a hernial sac contains omentum, he cannot tell that it does not contain intestine besides; secondly, a patient may die of strangulated omental hernia alone. I operated in the Middlesex Hospital upon an old man with strangulated omental hernia, there was vomiting, hiccup, and tenderness of the belly: h' sank, and died of peritoneal inflammation, which had made progress before the operation was performed.

When the strangulated hernia is merely omental, the patient has not so much pain and distress about the belly and the tumour, as in intestinal hernia; the symptoms are slower in their progress; the bowels are easily unloaded by enemata; the patient is then sensibly relieved; and opening medicines administered by the mouth complete the recovery.

The operation for strangulated hernia is the most difficult and the nicest in the compass of surgery: so possible is it in unusual cases (and no two cases in hernia are alike,) to go too far, or not to go far enough; *to injure the intestine, or not to open the sac, or not to divide the stricture.*

If the integument and subcutaneous layers of fascia are unusually thin, and the intestine in the sac is distended with flatus, it may be cut into in the first incision. I have seen this accident happen. It is not attended with any danger to the patient, who may almost be viewed in a state of greater security, through the temporary and immediate relief of the distension of the bowels through the wound. If the wound is a puncture merely, it may be secured with a knot of fine silk, the ends cut close off. When larger, I have seen the intestine returned all but the cut part, which is to be fastened by a suture to the integuments. The artificial anus closes in from six to eight weeks.

If the sac contains no water, either because none has been formed, or that it has been pressed into the abdomen, and the surface of the bowel has lost its polish from slight effusion of lymph, the surgeon may be in doubt whether he has yet reached the sac, when he has already divided it. I have seen the peritoneal coat of the intestine punctured in these circumstances, in the expectation that it would prove the sac.

I met with, in the dissecting-room, an old hernia, which appeared to have no sac. On the opposite side there was an ordinary hernia. A model of the appearance presented is in the museum of King's College. It appeared to me that there had been a hernial sac, the identity of which was lost, through its having become uniformly adherent to the intestine which it contained. If such a case occurred in practice, the surgeon would be almost excusable if he opened the intestine without suspecting it.

The cases are more various in which a surgeon may be nearly betrayed into not opening the hernial sac, or be led to think that he has done so, when he is yet without it.

It happens occasionally in the chapter of accidents, that an old hernial sac lies contiguous to that which contains the recent and strangulated hernia. An appearance similar to this is sometimes produced through condensation of the filamentous tissue of the groin by pressure. Layers of membrane closely resembling peritoneum are liable to be thus formed: I have, however, seen this appearance in crural hernia alone.

I recollect a case, in which a sac of this kind was supposed to be the hernial sac, which it contained; and the ligament of Gimbernat was divided, before the mistake was discovered. This mistake is the more easily made, that in such a case the finger introduced within the factitious sac passes under the crural arch, and seems to be contained in the narrow neck of a true sac.

A circumstance, which, when it occurs, contributes to strengthen this deception, is, that the serous cyst occasionally contains *liquid, the absence of which could otherwise strongly excite the suspicions of the surgeon that he had not yet opened the sac.* It has not happened to me to meet with an instance where the whole hernial sac has been contained in such a false sac; but I have known a false sac, attached to the outside of a hernial sac, convey the idea that a hernia of the head of the colon, with the characteristic partial sac, presented.

There is a complication of these serous sacs with condensed adipose tissue, which at first completely deceived me. In the first case which I witnessed of this description, the hernia was a crural hernia, and extremely small. After dividing several layers of fascia, I exposed a membrane resembling a hernial sac: upon opening it, I came upon what I conceived to be a nodule of omentum. This I cut through, in the expectation of finding intestine contained within it; instead of this, I came upon the true hernial sac, of remarkably small dimensions, and which I had not before reached. There was strangulated bowel within it.

It is my opinion, that, in the operation for strangulated hernia, *there is no safety for the patient, unless the hernial sac is opened.* In the first place, it is impossible, without this precaution, to tell with accuracy the condition of the bowel. The surgeon who has not inspected the strangulated intestine is in danger of returning it either mortified, or along with it some unsuspected accidental cause of stricture which was within the sac.

Secondly, although that part of the intestine which is loose in the sac may be in a state fit to be returned, yet that part may not be so, which is immediately compressed by the stricture. It is in general a prudent precaution to draw down the portion of intestine which has been nipped by the stricture, and to examine it, before returning the hernia. I have seen a patient die in consequence of this precaution being omitted. In the case I refer to all the bowel was sound, except the line actually constricted, which had mortified.

Thirdly, the stricture is very commonly either the neck of the sac itself, or so incorporated with it that it cannot be divided unless the serous membrane be divided too.

*But it is possible that the hernial sac may be properly opened, and yet the neck of the sac escape division.*

It is difficult to conceive how the following accident should arise, but I have seen a case in which it had actually happened. The case was strangulated crural hernia in a man. The surgeon laid open the sac: the gut was already mortified: there was a question of returning it; but it was necessary to divide the stricture, in order to allow of the free escape of the contents of the bowel at the wound. The surgeon divided what he supposed to be the stricture. The following day nothing had escaped through the wound, and when the finger was passed towards the belly from the cavity of the mortified intestine, it was found that there was no passage; the stricture still existed. Upon carefully examining the wound, it was found that the division of Gimbernat's and Poupart's ligament had been made external to the sac. Upon then dividing the true neck of the sac, the finger could be passed into the sound intestine within.

The case is more likely to happen, although it is an extremely rare case, in which the surgeon may have to operate upon a strangulated hernia which has already been *half forced back, sac and all,* within the abdominal parietes. In this case, it is obvious that, upon opening the hernial sac, and passing the finger towards its neck, the surgeon will feel the narrow ring in the abdominal walls at which the protrusion took place: this he will probably be satisfied with enlarging by a slight division; and, unless he is singularly circumspect, and has been quite alive to all the previous circumstances of the case, he may leave within the abdominal parietes an *undivided peritoneal stricture.*

One of the finest questions which occur in the operation for strangulated hernia, regards the degree of discolouration of the intestine at which it is unsafe to return it into the belly. But, in truth, it is not the colour of the intestine alone by which the surgeon ought, on this occasion, to be guided: he must likewise take into consideration the *quantity* of discoloured intestine; the *age* of the patient; the condition of the bowels within the abdomen; the length of time the strangulation has existed. I have seen patients die at different periods, from two to seven days, after the operation for strangulated hernia, in whom, upon a *post-mortem* examination, the intestine that had been returned has been found either to have mortified subsequently to its replacement, or not to have recovered its colour from the dark and suspicious hue it presented in the operation. These patients have died of peritonitis; and, although several would in all probability have died if the unhealthy intestine had not been returned into the belly, I am convinced that others would have lived, if, instead of the whole, the healthiest part only of the protruded bowel had been reduced, and the most discoloured part opened, and retained in the sac. In a small proportion of these serious cases the scale is probably turned against the patient by the bowel being called upon, and being unable, at once, to recover its healthy state, and to continue its functions. Relieve it by a direct outlet of its contents, and the chance of its self-recovery ought to be, and, I think I have found it so practically, is considerably increased. The artificial anus is of no consequence: it will close in a few weeks. This important question, which has seldom to be discussed except in hospital practice, applies only to cases which have become aggravated through delay and neglect before a surgeon is consulted.

